# Systematic quantification and removal of host DNA contamination in 16S rRNA gene sequencing

**DOI:** 10.1002/qub2.70055

**Published:** 2026-09-14

**Authors:** Till Birkner, Theda Ulrike Patricia Bartolomaeus, Victoria McParland, Sofia Kirke Forslund‐Startceva, Ulrike Löber

**Affiliations:** ^1^ Host‐Microbiome Factors in Cardiovascular Disease Lab Max Delbrück Center for Molecular Medicine in the Helmholtz Association Berlin Germany; ^2^ Experimental and Clinical Research Center A Cooperation of Charité – Universitätsmedizin Berlin and Max Delbrück Center for Molecular Medicine Berlin Germany; ^3^ Charité – Universitätsmedizin Berlin Corporate Member of Freie Universität Berlin Humboldt‐Universität zu Berlin and Berlin Institute of Health Berlin Germany; ^4^ German Centre for Cardiovascular Research Partner Site Berlin Berlin Germany; ^5^ Department of Bioscience Centre for Ecological and Evolutionary Synthesis (CEES) University of Oslo Oslo Norway; ^6^ Berlin Institute of Health (BIH) Berlin Germany; ^7^ European Molecular Biology Laboratory, Structural and Computational Biology Unit Heidelberg Germany

**Keywords:** 16S rRNA gene sequencing, host contamination, low‐biomass samples, microbiome quality control, microbiome standards, off‐target amplification

## Abstract

16S ribosomal RNA (rRNA) gene sequencing is a standard tool for microbial community analysis. Challenges can occur, particularly in low‐biomass samples, when host DNA triggers off‐target amplification. Low‐biomass microbiome studies are particularly vulnerable to contamination from host DNA, which can obscure microbial signals and bias interpretation. This contamination presents a significant barrier to accurately characterizing microbial communities, especially in clinical or environmental samples with limited bacterial DNA. To systematically quantify and mitigate host DNA interference, we constructed a bacterial mock community dilution series spiked with controlled proportions of human DNA. Using 16S rRNA gene sequencing, we assessed how increasing host DNA affects microbial community profiles and evaluated several computational approaches for removing host‐derived sequences, including pre‐clustering filtering, post‐clustering operational taxonomic unit (OTU) filtering, and the R package Decontam. We found that off‐target amplification was more prevalent when the bacterial content was less than 10% relative to host DNA. Total DNA concentration induced minimal bias. Post‐clustering OTU filtering and reference genome mapping effectively reduced host contamination. Among the tested correction strategies, post‐clustering OTU filtering proved most effective and computationally sustainable, achieving nearly complete removal of host‐derived reads with minimal effect on microbial diversity estimates. Although 16S rRNA gene sequencing remains a cost‐effective and high‐throughput technology, it requires rigorous methodological controls in low‐biomass contexts. Our study offers a systematic evaluation of off‐target amplification effects and practical mitigation strategies to improve the accuracy of microbial community analysis. The presented framework provides a robust and scalable approach for identifying and removing host contamination from low‐biomass 16S rRNA sequencing data.

## 1 INTRODUCTION

High‐throughput sequencing has transformed our ability to profile microbial communities and other complex biological systems. However, sequencing data from low‐biomass samples remains especially vulnerable to bias [1]. A significant challenge arises when host DNA dominates the nucleic acid pool, leading to off‐target amplification and distortion of microbial profiles [2]. This problem is not unique to microbiome studies; it represents a broader issue in nucleic acid research, where contaminating or mis‐amplified sequences can obscure genuine biological signals.

16S ribosomal RNA (16S rRNA) gene sequencing remains widely used for microbial profiling due to its cost‐effectiveness and targeted recovery of microbial signatures from limited sample material. Yet, in low‐biomass contexts, host DNA contamination introduces systematic artifacts that compromise accuracy and reproducibility [3]. Existing computational strategies for contamination removal have not been rigorously benchmarked under controlled conditions, leaving their effectiveness and scalability uncertain [4].

Recent advancements in microbiome research have highlighted the critical role of microbial communities in human health, especially in less accessible ecosystems such as tissue [5, 6], blood [7], and tumor [8] microbiomes. These high‐profile studies underscore the importance of understanding these communities and highlight the challenges posed by low‐biomass samples. In such samples, the risk of contamination and artifact generation can significantly distort outcomes [9, 10, 11, 12, 13]. This is evident, for example, in studies investigating cancer or brain microbiome, highlighting the need for careful methodological refinement to avoid misleading conclusions that could impact research directions and clinical applications [13, 14].

Here, we present a systematic framework for quantifying the impact of host DNA contamination and evaluating computational strategies to mitigate it. Using a bacterial mock community dilution series with controlled proportions of human DNA, we assess how host contamination affects microbial profiles, identify thresholds at which contamination dominates, and benchmark multiple removal strategies. We further evaluate the computational performance of different approaches, focusing on scalability and practical applicability. Our study aims to contribute to the development of methodological standards for sequencing‐based analyses in low‐biomass contexts.

In this context, 16S rRNA gene sequencing remains a valuable tool due to its cost‐effectiveness and adaptability [15, 16], but it is particularly advantageous for low‐biomass samples. Unlike untargeted shotgun sequencing, which requires impractically deep sequencing and often results in discarding most of the data, 16S rRNA sequencing utilizes targeted amplification to retrieve microbial signatures even when DNA is scarce [3]. However, this technique is not without its biases. Systematic distortions can occur at various stages of the sequencing workflow, from cell lysis to bioinformatic analysis [3, 17, 18, 19]. These biases necessitate rigorous controls and highlight the limitations of comparing results across different protocols [20, 21].

Our study builds on these insights and explicitly addresses the contamination challenges inherent in 16S rRNA gene sequencing of low‐biomass samples [3, 17, 18, 19]. These challenges were evident in our examination of sputum samples from human immunodeficiency virus (HIV)‐infected individuals with chronic obstructive pulmonary disease (COPD) in rural Uganda, where we observed significant human DNA contamination, particularly pronounced in the COPD+ group. If left uncorrected, this contamination could be misconstrued as microbial data [22]. This realization forms the basis of our current research, which uses a bacterial mock community dilution series spiked with varying amounts of human DNA to systematically investigate the extent of host contamination and its impact on microbial profile accuracy.

We investigated whether thresholds exist at which host DNA contamination dominates sequencing output. We demonstrated that post‐clustering operational taxonomic unit (OTU) filtering and reference genome mapping effectively mitigate these biases. These techniques are critical for improving the reliability of microbial community analyses in clinical and environmental settings characterized by low biomass.

In summary, whereas 16S rRNA gene sequencing remains an indispensable tool for microbial community analysis, particularly in clinical contexts involving low‐biomass samples, it necessitates stringent methodologies and robust controls to ensure accurate characterization [12, 15, 16, 23]. Our work highlights these challenges and offers practical solutions to enhance precision in microbiome studies at the frontier of low‐biomass research. By effectively addressing host DNA contamination, we emphasize the crucial importance of methodological rigor in understanding the role of microbial communities in health and disease.

## 2 RESULTS

### 2.1 Composition analysis of bacterial mock community and control samples

The 16S rRNA gene sequencing data analysis revealed that even the pure bacterial mock community did not perfectly align with its theoretical composition (Figure 1A,B; Bray–Curtis dissimilarity between pure bacterial sample and the theoretical community composition = 0.35). Individual sample‐level variability underlying this comparison is shown in Supporting Information S1: Figure S1. In contrast, other experimental samples exhibited community compositions more closely resembling the sequenced pure bacterial mock community (Figure 1B), with Bray–Curtis dissimilarities ranging from 0.01 to 0.17 (mean = 0.04, standard deviation = 0.03). The experimental contaminant control sample (DNase/RNase‐free H_2_O) was predominantly composed of the genus *Cutibacterium* (259 of 265 total reads). Similarly, in a pure human DNA sample without any bacterial spike‐in, 39 of 4457 total reads were assigned to bacterial taxa using the SILVA 138 database [24], of which 30 were classified as *Cutibacterium*. The consistent detection of *Cutibacterium* (formerly *Propionibacterium*) in negative controls and human‐only samples is consistent with known background contamination from laboratory reagents and handling, particularly in low‐biomass workflows. This genus is frequently reported as a common contaminant in microbiome studies and should therefore be interpreted with caution [1, 25, 26].

**FIGURE 1:**
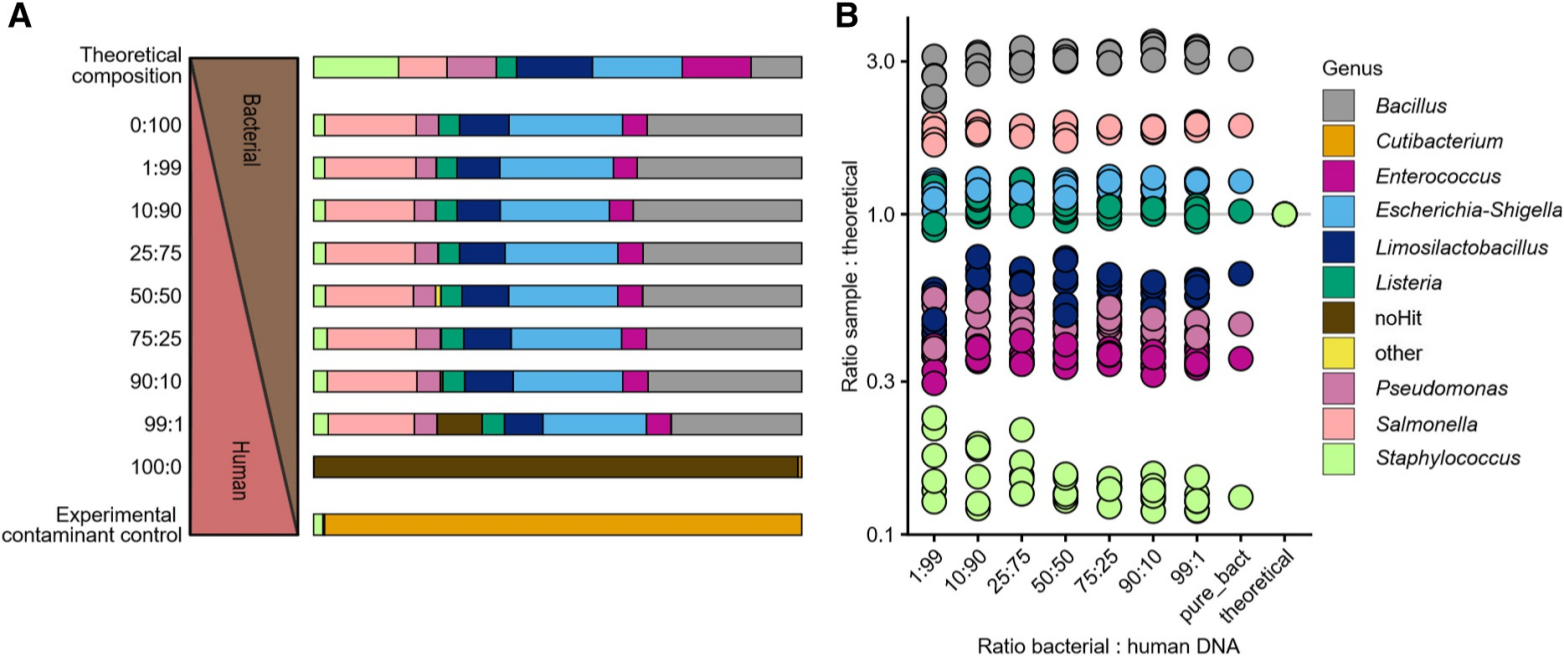
Analysis of microbial community composition and experimental controls. The colors correspond to different bacterial genera, as indicated in the legend. (A) Relative abundance of bacterial genera: theoretical composition of microbial communities and varying ratios of bacterial to human DNA. Each bar represents a different ratio, ranging from 0:100 (pure human DNA) to 100:0 (pure bacterial DNA = pure_bact). (B) Ratio of sample to theoretical values: Each dot represents a genus, with its position along the *y*‐axis indicating the difference between the observed microbial composition of the sample and theoretical values. The *x*‐axis corresponds to the different bacterial‐to‐human DNA mixtures; multiple dots of the same color represent the same ratio within the dilution series. The horizontal line at a ratio of 1.0 serves as a reference point where the sample matches the theoretical expectation.

### 2.2 Host DNA contamination predominance in low biomass samples

In samples with elevated proportions of human DNA relative to bacterial mock DNA, there was a notable overrepresentation of sequences that did not align with any entries in the SILVA 16S rRNA gene database. When human DNA accounted for more than 90% of the total DNA content, off‐target amplification of host DNA became a significant issue, introducing biases that obscured bacterial signals. Using BLASTn [27] to query the sequences against the NCBI nucleotide database (nt) [28], we found that most sequences showed high identity to human chromosome 16. Lower‐identity matches to other primate sequences likely reflect highly conserved genomic regions shared with human DNA.

Besides sequences identified as originating from *Homo sapiens*, a notable proportion was associated with synthetic eukaryotic constructs, suggesting the presence of laboratory artifacts or engineered sequences commonly used in research. Additionally, a few sequences matched *Pongo abelii* and *Pan troglodytes*, further supporting the hypothesis of conserved primate DNA regions. Interestingly, a subset of sequences was also identified as originating from *Takifugu rubripes* (Japanese pufferfish), indicating potential cross‐contamination or database artifacts. These patterns are summarized in Figure 2, which shows the proportion of raw, human‐associated, and filtered reads across samples (Figure 2A), the log‐ratio of bacterial to human reads (Figure 2B), and corresponding DNA concentrations (Figure 2C).

**FIGURE 2:**
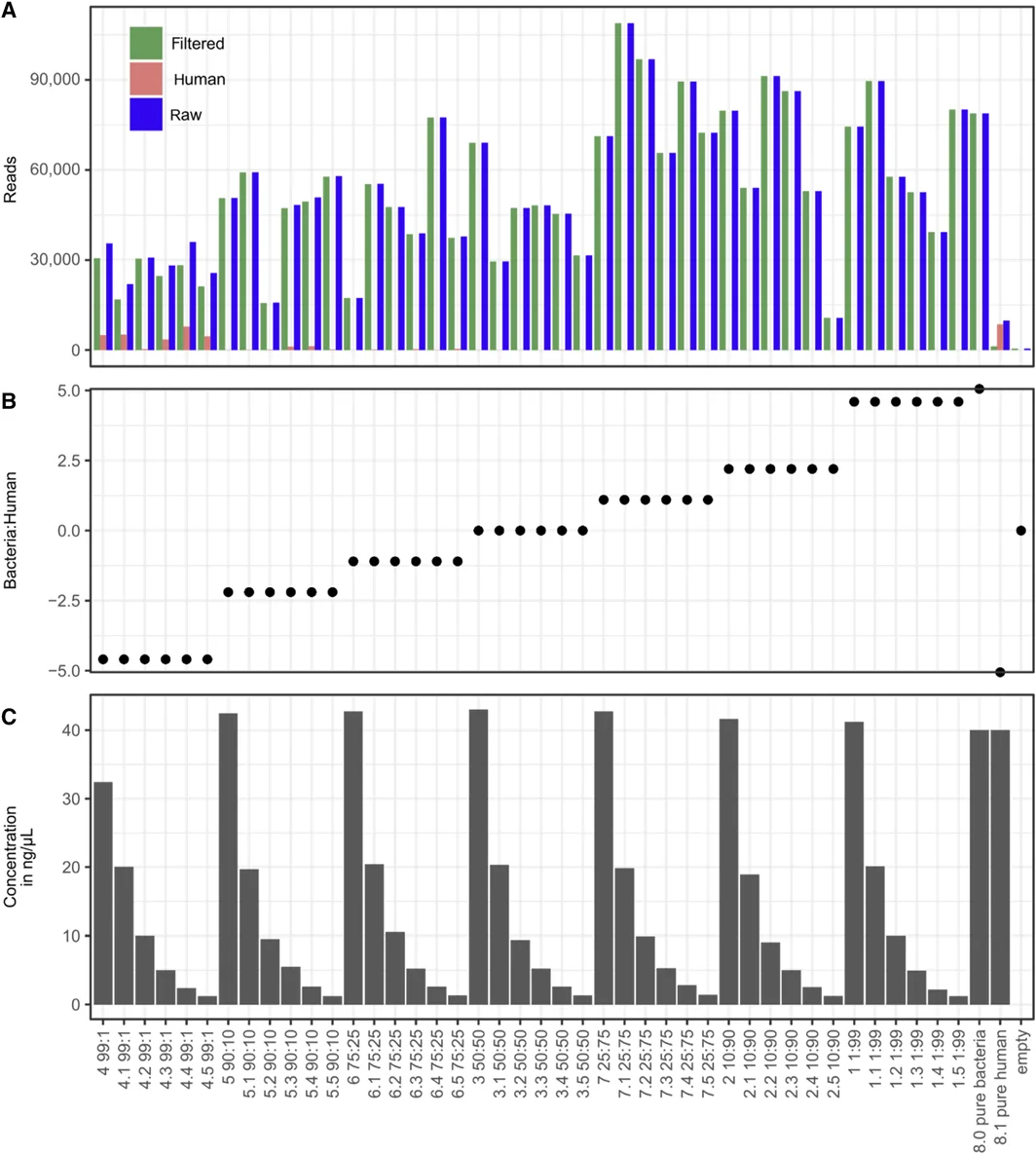
Comparative analysis of sequencing reads across different samples under three data processing conditions: raw, human, and filtered. (A) Total number of reads for each sample, with raw data indicated by blue bars, human‐associated reads by red bars, and filtered data, which likely represents non‐human reads, by green bars. (B) The log‐ratio of bacterial to human reads. (C) The DNA concentration (ng/µL) for the bacterial and human reads.

### 2.3 Relative non‐bacterial DNA content is more important than absolute DNA concentration

We designed a bacterial mock community dilution series to investigate the relationship between human DNA content and off‐target amplification. As the proportion of human DNA increased, the number of 16S rRNA gene sequences misattributed to the host genome also increased. However, the overall DNA concentration had minimal impact on the Bray–Curtis dissimilarity index (Table 1).

**TABLE 1:** Permutational multivariate analysis of variance (PERMANOVA) analysis of beta diversity in microbial communities.

	SumOfSqs	*R* ^2^	*F*	Pr (>*F*)	Sign.
Human DNA	0.007797	0.19631	10.7945	0.001	***
Concentration	0.003029	0.07626	4.1931	0.008	**
Residual	0.028892	0.72744			
Total	0.039717	1.00000			

*Note*: *** for *p* < 0.001, ** for *p* < 0.01.

Abbreviations: *F*, *F*‐statistic; Pr (>*F*), *p*‐value; sign, statistical significance; SumOfSqs, the sum of squares.

Principal coordinates analysis (PCoA) further corroborated these findings, revealing distinct clustering of microbial communities driven primarily by the ratio of bacterial to human DNA rather than the total DNA concentration (Figure 3). Notably, the “pure_bact” sample, which contained only bacterial DNA, exhibited minimal variation and was distributed across a narrow range in the ordination plot. This suggests that the absence of human DNA reduces variability in community composition across different DNA concentrations.

**FIGURE 3:**
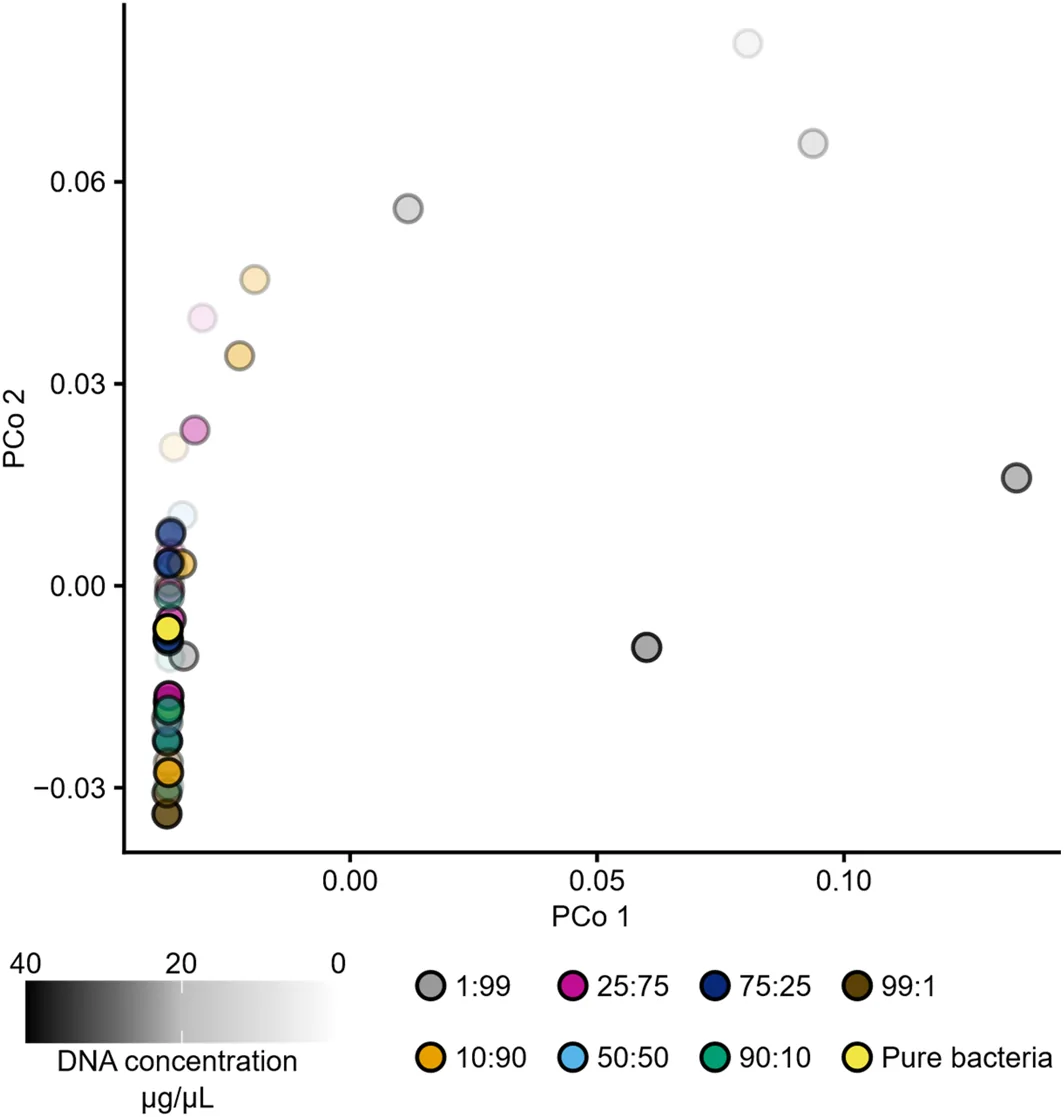
Principal coordinates analysis (PCoA) of microbial communities by bacterial‐to‐human cell ratios: PCoA compares microbial community structures across samples with varying ratios of bacterial to human DNA. The color of each data point indicates the specific bacterial‐to‐human DNA ratio, ranging from predominantly human (1:99) to predominantly bacterial (99:1), with a category for pure bacterial samples (pure_bact). The opacity correlates with the overall DNA concentration of a particular sample, with paler samples being more diluted.

Permutational multivariate analysis of variance (PERMANOVA) analysis [29] was used to evaluate the impact of human DNA content and total DNA concentration on the beta diversity of microbial communities, quantified using the Bray–Curtis dissimilarity index. The table summarizes the sum of squares (SumOfSqs), the proportion of variance explained (*R* ^2^), *F*‐statistic (*F*), *p*‐value (Pr (>*F*)), and statistical significance (sign). The analysis reveals that human DNA content significantly influences community composition (*R* ^2^ = 0.20, *p*‐value = 0.001), followed by total DNA concentration (*R* ^2^ = 0.08, *p*‐value = 0.008), which accounts for a significant portion of the variance in microbial diversity. However, 73% of the variation remains unexplained (residuals, *R* ^2^ = 0.73). Statistical significance is denoted as *** for *p* < 0.001, ** for *p* < 0.01, and * for *p* < 0.1. Absolute taxon abundances across DNA concentration and bacteria‐to‐human DNA ratio, before and after contamination filtering, are shown in Supporting Information S1: Figure S2, confirming that relative host DNA content rather than total DNA concentration primarily drives compositional shifts.

As the proportion of human DNA increases, the relative abundances of *Bacillus*, *Escherichia‐Shigella*, *Salmonella*, and *Cutibacterium* significantly decrease (Figure 4). Concurrently, the abundance of non‐bacterial reads classified as “noHit” increases substantially, indicating that higher proportions of human DNA lead to more off‐target amplicons. In contrast, total DNA concentration positively correlates with the abundance of *Listeria*, *Staphylococcus*, *Enterococcus*, and *Pseudomonas* (Figure 4). The genera *Bacillus*, *Escherichia‐Shigella*, and *Salmonella* appear less affected by changes in total DNA concentration compared to the percentage of human DNA.

**FIGURE 4:**
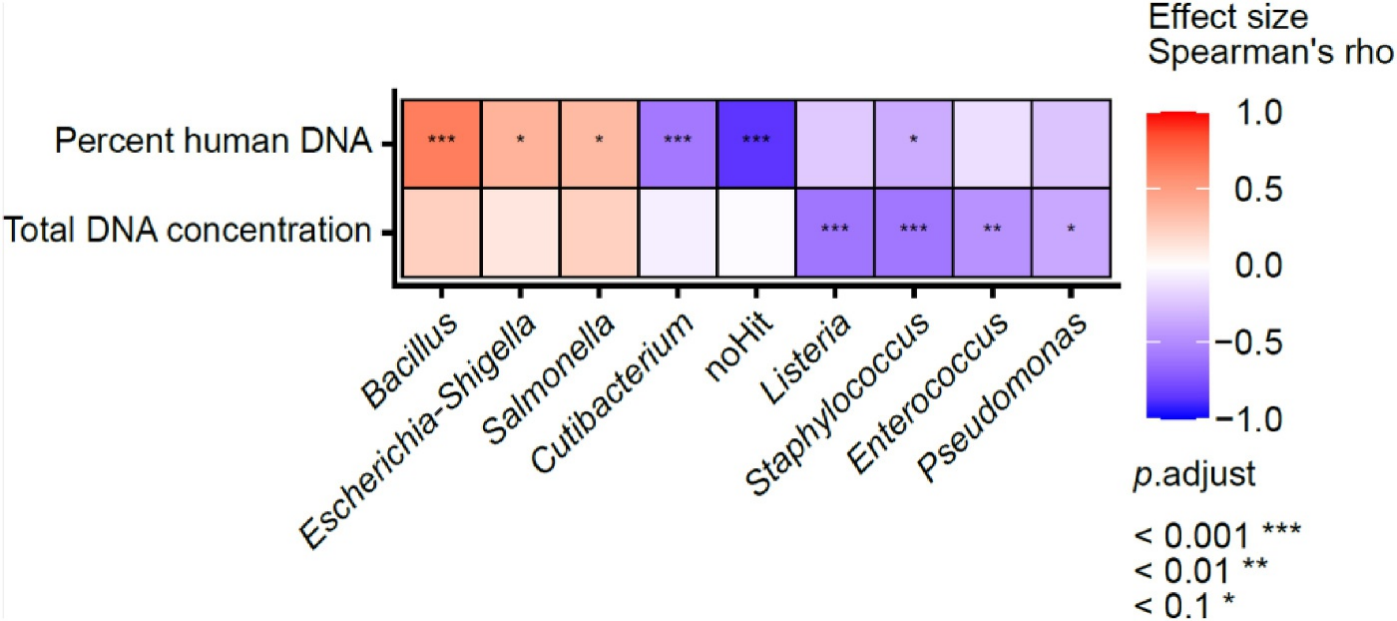
Bacterial genera differential abundance (effect size Spearman’s rho) in response to two variables: percent human DNA and total DNA concentration. A positive value (red) indicates a genus is more abundant, and a negative value (blue) means it is less abundant. Asterisks indicate statistical significance of Spearma’s correlation tests after multiple testing correction with the Benjamini–Hochberg procedure (*q* < 0.1, *q* < 0.01, *q* < 0.001). Their vertical position within tiles is for visualization purposes only and does not encode additional information.

A substantial proportion of the variance in microbial community composition remained unexplained in the PERMANOVA analysis. This residual variance likely reflects a combination of factors inherent to 16S rRNA gene sequencing, including polymerase chain reaction (PCR) amplification bias, stochastic variation in low‐biomass samples, and differences in sequencing depth. In low‐biomass contexts, small fluctuations in absolute DNA quantities can disproportionately influence relative abundance estimates, further increasing variability. Additionally, compositional effects and genus‐specific amplification efficiencies may contribute to the observed variation, particularly with respect to total DNA concentration. These factors highlight the inherent complexity of interpreting microbiome data in low‐biomass settings.

In summary, higher percentages of human DNA reduce the detectability of specific bacterial taxa and increase the proportion of unidentified sequences. In contrast, higher total DNA concentrations enhance the detectability of particular genera, such as *Listeria* and *Staphylococcus*.

### 2.4 Efficacy of contamination removal methods in 16S rRNA gene sequencing data

We evaluated three computational methods for removing contamination from 16S rRNA gene sequencing data: a pre‐clustering removal approach [30], a post‐clustering removal approach (LotuS2) [31], and the R package Decontam [32]. We compared these methods against unfiltered (naive) data. Our analysis revealed that the Decontam package was less effective at filtering host contamination in low‐biomass samples (Supporting Information S1: Figure S3), potentially because it is not optimized for such datasets (as discussed).

Figure 5 and Table 2 summarize the relative abundances of non‐bacterial sequences remaining after each method was applied, highlighting their comparative effectiveness in mitigating human DNA contamination and cleaning the dataset.

Lower percentages of “noHit” reads correspond to more effective contamination removal. Sequences classified as “noHit” were interpreted primarily as off‐target amplification products or host‐derived sequences in this controlled mock community setting. Whereas such reads may in principle include unannotated microbial sequences, this is unlikely in the present study. Taxonomic assignment was performed using permissive thresholds as low as 78% identity at the phylum level, yet these sequences remained unclassified, indicating no detectable similarity to known microbial reference sequences. This strongly supports the interpretation that “noHit” reads predominantly reflect non‐microbial or off‐target amplification products. However, in more complex environmental or clinical samples, where unknown taxa are more prevalent, this assumption may not fully hold and should be interpreted with caution.

**FIGURE 5:**
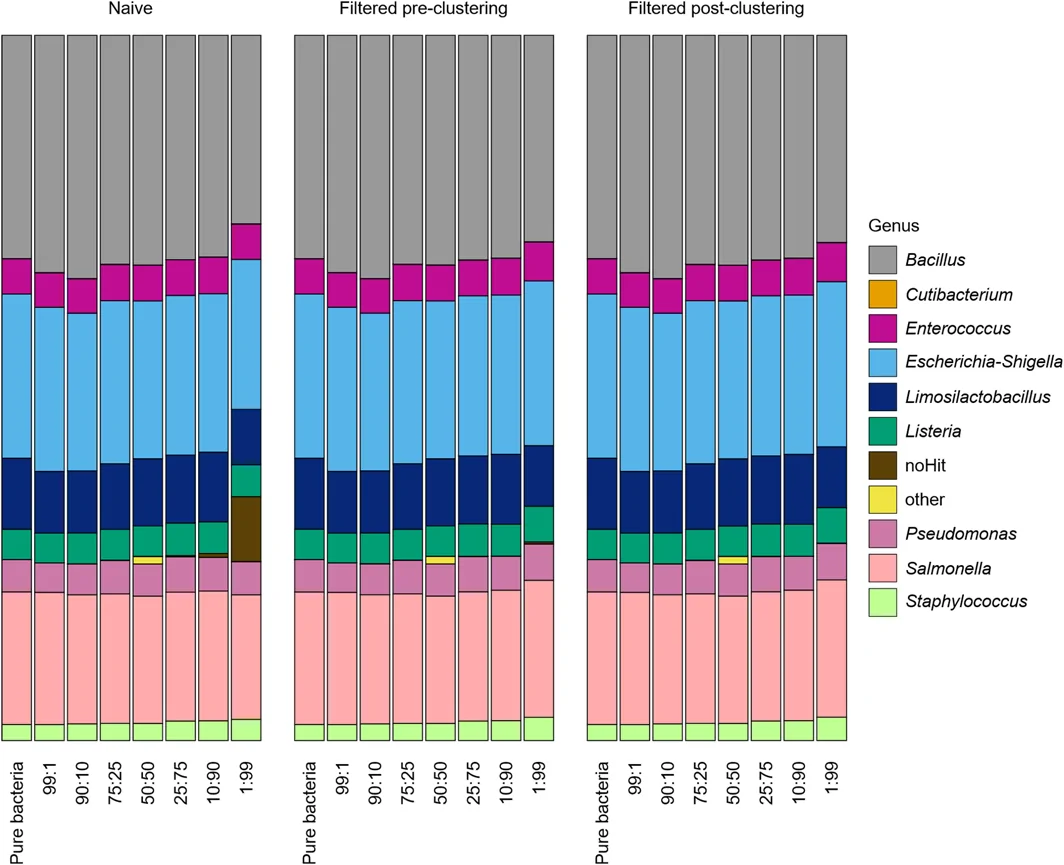
Relative abundance of genera grouped by bacterial‐to‐human DNA ratio for naive (left), pre‐clustering (middle), and post‐clustering (right) filtered dataset. Each column, except “pure bact”, depicts the mean relative abundance of the dilution series of the respective ratio.

**TABLE 2:** Cumulative amount of “noHit” reads determines reads not mapped to any of the references in the SILVA 16S rRNA database (v138).

Variable	Mix b/h	Raw	Pre_c	Post_c	Decontam_R
noHit	empty	0.00000%	0.00000%	0.000000%	0.00000%
noHit	pure_bact	0.00276%	0.00276%	0.002765%	0.00276%
noHit	99:1	0.00078%	0.00078%	0.000782%	0.00078%
noHit	75:25	0.00662%	0.00309%	0.002885%	0.00536%
noHit	90:10	0.00765%	0.00516%	0.005160%	0.00745%
noHit	50:50	0.02004%	0.00346%	0.002796%	0.01659%
noHit	25:75	0.17663%	0.00556%	0.001179%	0.13525%
noHit	10:90	0.53035%	0.02004%	0.003628%	0.41256%
noHit	1:99	9.19731%	0.29736%	0.005643%	6.90936%
noHit	pure_human	99.12497%	75.47170%	0.000000%	98.89362%

*Note*: “Pure_human” denotes the sample with only human DNA, and “pure_bact” denotes the sample with only bacterial DNA. The percentages are derived from the relative abundance of non‐bacterial sequences after each filtering step, indicating the proportion of remaining contamination.

Abbreviations: Decontam_R, using the Decontam package; Post_c, post‐clustering filtering; Pre_c, pre‐clustering filtering.

### 2.5 Validation using ddPCR—16S rRNA gene amplification

In this study, we employed a TaqMan assay in digital droplet PCR (ddPCR) to quantify the absolute copy numbers of 18 and 16S rRNA genes in our samples. The primary aim was to validate the ratios used to assess human contamination in the 16S rRNA gene data. Absolute copy numbers were calculated by multiplying the ddPCR output concentration (copies/µL) by the sample‐specific dilution factor.

Because of inherent differences in ribosomal gene copy numbers per unit of DNA resulting from variations in genome size, we normalized the values to achieve equality for the 50:50 ratio sample (Figure 6).

**FIGURE 6:**
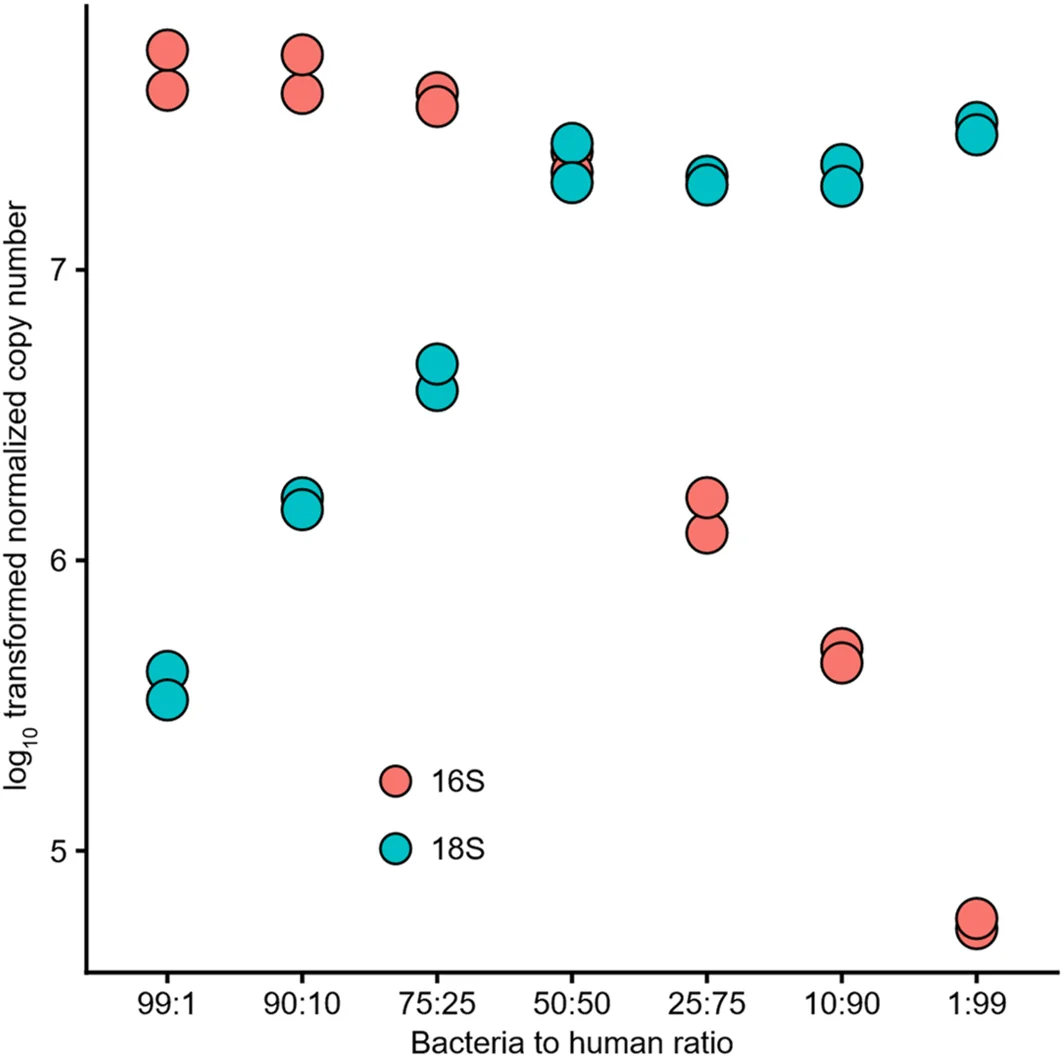
Microbial (16S) and eukaryotic (18S) small ribosomal subunit gene copy numbers of samples with varying ratios of bacterial‐to‐human DNA, based on digital droplet PCR (ddPCR). Copy numbers are normalized to equal 16 and 18S at a 50:50 ratio in the sample and then log‐transformed.

## 3 DISCUSSION

The analysis conducted in this study addresses three critical hypotheses regarding the influence of off‐target human DNA amplification and contamination in samples with low microbial biomass. The findings of our investigation into 16S rRNA gene sequencing data highlight the inherent biases introduced by this sequencing approach in low‐biomass samples, even when applied to a pure bacterial mock community. As evidenced by discrepancies between observed and theoretical microbial compositions, these biases highlight the challenges of accurately characterizing microbial community structure [10, 11, 21]. Such biases are consistent with prior findings, demonstrating that 16S rRNA sequencing is prone to systematic errors introduced at various workflow stages, including DNA extraction, PCR amplification, and sequencing [10, 11, 21]. These findings significantly contribute to understanding the complexities of microbial community analysis and to developing methods for removing contamination.

This analysis explored strategies to mitigate host DNA contamination, focusing on filtering reads that align to the reference genome before and after OTU clustering. Both methods effectively removed contaminants; however, post‐clustering OTU filtering proved more efficient than reference genome mapping, which was computationally intensive yet less effective. This finding aligns with the growing emphasis on sustainable research practices. By conserving computational resources, post‐clustering removal reduces the carbon footprint associated with large‐scale genomic analyses and offers a practical solution for routine microbiome studies [33]. These practical implications make the research directly relevant to the daily work of researchers and professionals in the field.

Additionally, we evaluated the performance of the Decontam package for identifying and removing contamination in low‐biomass samples. Our results revealed limitations in its efficacy under these conditions. The statistical models employed by Decontam, particularly those relying on an inverse relationship between contaminant frequency and sample biomass, were less reliable in contexts with low biomass. Low biomass samples inherently have a higher risk of contamination, and their dilute nature can result in contaminant DNA being disproportionately overrepresented. Furthermore, Decontam’s dependency on negative control samples and DNA quantitation data posed significant challenges due to the variability and low DNA yields characteristic of samples with low biomass. These findings underscore the need for further exploration and innovation in contamination identification and removal methods in low‐biomass contexts.

The observed differences between contamination removal strategies can be explained by their underlying methodological assumptions. Pre‐clustering filtering relies on read‐level alignment to a reference genome, which can remove ambiguous sequences but may also inadvertently discard true microbial reads that are partially similar to host DNA. In contrast, post‐clustering filtering operates at the OTU level, allowing sequence clustering to first capture biological variation before removing OTUs that match off‐target references. This approach is more robust to noise and reduces the risk of over‐filtering.

Statistical approaches such as Decontam rely on assumptions about the relationship between contaminant abundance and sample characteristics, such as DNA concentration or prevalence in negative controls. Although effective in higher‐biomass settings, these assumptions may break down in low‐biomass samples, where stochastic effects and high levels of relative contamination obscure the expected patterns. These conceptual differences explain the reduced performance of Decontam observed in our study. These findings are likely generalizable to other contamination‐removal strategies that rely on similar principles. Methods based on sequence alignment may face similar trade‐offs between sensitivity and specificity, whereas statistical approaches may be similarly challenged by the altered signal‐to‐noise ratio in low‐biomass datasets. This highlights the need for careful method selection depending on sample type and study design.

Our analysis also identified unexpected contaminants (e.g., *Vagococcus, Cetobacterium*, *Aeromonas, Vibrio,* and *Plesiomonas*) in our 50:50 mixture at the lowest dilution. These contaminants likely originated from laboratory environments or reagents, emphasizing the susceptibility of highly diluted samples to external contamination. This finding underscores the crucial need for rigorous quality control measures in microbiome studies, including technical and biological replicates, to identify and mitigate contamination sources effectively. It also highlights the potential for unexpected contaminants in microbiome studies, which researchers should be aware of when interpreting their results.

Furthermore, our investigation highlights the pervasive issue of database mal‐annotation, as previously reported by Orakov et al. [34]. The fidelity of microbial databases is increased when rigorous protocols for remediating contamination are integrated into analysis pipelines. Tools like GUNC [34] have revealed that approximately 5.7% of genomes in GenBank and 5.2% in the NCBI reference sequence database (RefSeq) are contaminated chimeras, alongside 15%–30% of “high‐quality” metagenome‐assembled genomes from recent studies. Such contamination estimates distort diversity estimates, functional annotations, and downstream biological inferences. This issue is particularly problematic for low biomass samples, where contaminant DNA can overshadow genuine microbial signals, leading to overestimations of diversity and erroneous conclusions about microbial ecology and evolution [21, 34].

Taken together, our findings demonstrate that DNA concentration is less critical than the presence of biomass contamination in influencing sequencing outcomes. We demonstrate that post‐clustering OTU filtering effectively addresses this issue, representing a computationally sustainable solution for removing off‐target amplification. Based on these findings, we recommend using pipelines such as LotuS2 [31], which incorporates an integrated off‐target database to filter host contamination, particularly for low‐biomass samples, effectively.

In addition to computational approaches, experimental strategies such as hybrid capture, primer extension capture, single primer extension, or target enrichment offer an alternative means of reducing host DNA interference [35, 36]. These methods can selectively enrich microbial sequences prior to sequencing, thereby improving sensitivity in low‐biomass samples. However, they require prior probe design and may introduce biases similar to primer‐based amplification. Furthermore, their cost and complexity may limit their applicability in large‐scale studies. As such, computational approaches remain an important and flexible component of contamination mitigation strategies.

A limitation of this study is that contamination controls were limited to PCR‐ and reagent‐level controls and did not include full‐process controls spanning sample collection through sequencing. Although the controlled mock community design allows precise quantification of host DNA effects, future studies incorporating complete workflow controls would provide additional insights into contamination sources in real‐world samples.

## 4 CONCLUSION

Our study systematically demonstrates that host DNA contamination strongly biases 16S rRNA gene sequencing in low‐biomass samples, with off‐target amplification dominating when host DNA exceeds approximately 90% of total DNA. This threshold provides an empirical reference point for interpreting microbiome data derived from low‐biomass environments.

Among the evaluated approaches, post‐clustering OTU filtering emerged as the most effective and computationally efficient strategy for mitigating host‐derived signal without substantially distorting microbial community structure. In contrast, methods relying solely on statistical assumptions or pre‐clustering filtering showed reduced performance under low‐biomass conditions.

These findings have direct implications for the design and analysis of microbiome studies, particularly in clinical and environmental settings where host DNA is abundant. They highlight the importance of integrating both experimental controls and appropriate computational strategies to ensure reliable results. To our knowledge, this is the first systematic quantification of this threshold under controlled conditions.

Future work should explore how these findings extend to more complex real‐world microbiomes and alternative sequencing approaches, including enrichment‐based methods. Furthermore, additional experiments in the 90%–100% host DNA range could better characterize contamination effects. Overall, this study provides a practical framework for improving data quality and interpretability in low‐biomass microbiome research.

## 5 MATERIALS AND METHODS

### 5.1 Experimental design

The overall experimental design, including the dilution series and control samples, is illustrated in Figure 7.

**FIGURE 7:**
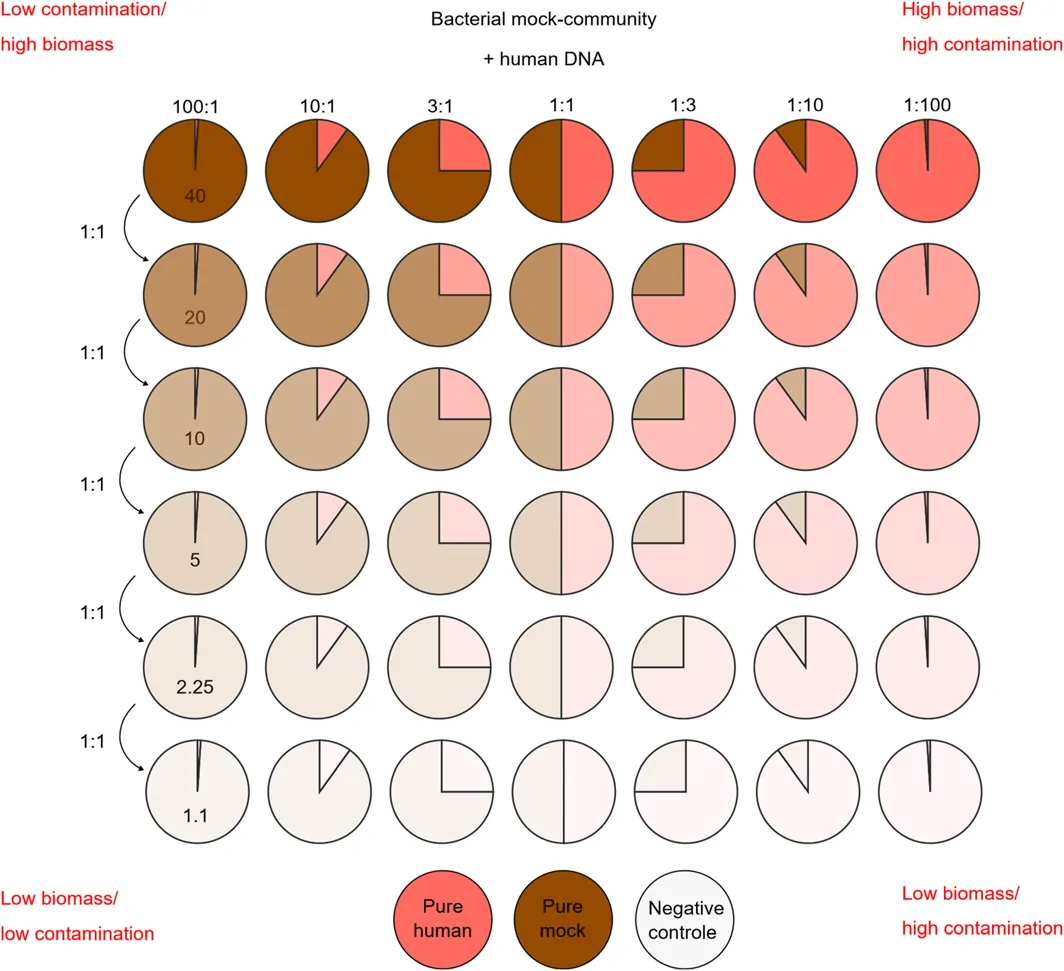
Experimental design. Various conditions are shown, with brown representing the ratio of bacterial DNA and pink indicating the ratio of human DNA. The transparency encodes for DNA concentration (dilution). Charts denote biomass and contamination levels, ranging from high biomass/low contamination to low biomass/high contamination. Controls for human, mock, and negative samples are included for reference.

### 5.2 DNA extraction and environmental contamination control

To minimize environmental contamination, all laboratory procedures were conducted under a laminar flow hood (LabGarda ES Energy Saver Class II Laminar Flow, NuAire Inc., Plymouth, MN, USA).

#### 5.2.1 Microbial standard preparation

The ZymoBIOMICS microbial community standard (Zymo Research Europe GmbH, Freiburg, Germany), comprising eight bacterial and two fungal strains, was used as the mock community in this study. The standard was stored at −20 °C until use. Before DNA extraction, the sample was thawed and vortexed to ensure uniform distribution of the microbial content.

#### 5.2.2 DNA extraction procedure

DNA extraction was performed using 75 μL of the microbial standard with the ZymoBIOMICS DNA Miniprep Kit, following the manufacturer’s protocol with slight modifications to enhance mechanical disruption. Samples were transferred to a PeQLab (Bertin Corp., Rockville, MD, USA) vial containing ZR BashingBead lysis tube beads (0.1 and 0.5 mm). The vial was securely capped after 750 μL of ZymoBIOMICS lysis solution was added.

Mechanical disruption was performed using a PeQLab Precellys 24 homogenizer (Bertin Corp., Rockville, MD, USA) at 5500 rpm for two 15‐s cycles, with a 5‐min rest between cycles. DNA was eluted using 50 μL of ZymoBIOMICS DNase/RNase‐free water.

The DNA extraction was conducted in eight independent rounds to ensure reproducibility and consistency. The DNA extracted from all rounds was pooled to achieve a uniform sample for downstream analyses. Human genomic DNA was obtained from PROMEGA Corporation (Madison, WI, USA; catalog reference G3041) and used without further modification. The concentrations of microbial and human genomic DNA standard samples were quantified using a Qubit fluorometer (Qubit 4, Thermo Fisher Scientific, Waltham, MA, USA). The measured concentrations were 170 ng/µL for the human genomic DNA and 52 ng/µL for the microbial standard.

#### 5.2.3 Preparation of DNA mixtures and dilution series

To simulate varying microbial‐to‐human DNA ratios, microbial and human DNA were mixed according to predefined proportions corresponding to sample names 1–7, as outlined in Table 3. Subsequently, the DNA mixtures were further diluted using DNase‐ and RNase‐free water to create a continuous dilution series, detailed in Table 3 under sample names 1.1–7.5. This dilution series was designed to investigate the effects of varying DNA ratios and concentrations on microbial detection and analysis outcomes.

**TABLE 3:** Dilution series.

Sample name	Sample identifier	Concentration	Human	Bacterial	H_2_O (µL)	Standard (µL)	Comment
1	P01‐A01‐1	41.2	1	99	NA	50	NA
2	P01‐B01‐2	41.6	10	90	NA	50	NA
3	P01‐C01‐3	43.0	50	50	NA	50	NA
4	P01‐D01‐4	32.4	99	1	NA	50	NA
5	P01‐E01‐5	42.4	90	10	NA	50	NA
6	P01‐F01‐6	42.7	75	25	NA	50	NA
7	P01‐G01‐7	42.7	25	75	NA	50	NA
8	P01‐H01‐8	40.0	0	100	NA	50	NA
1.1	P01‐A02–1.1	20.1	1	99	25	25	1:1 from 1
1.2	P01‐B02–1.2	10.0	1	99	25	25	1:1 from 1.1
1.3	P01‐C02–1.3	4.9	1	99	25	25	1:1 from 1.2
1.4	P01‐D02–1.4	2.2	1	99	25	25	1:1 from 1.3
1.5	P01‐E02–1.5	1.2	1	99	25	25	1:1 from 1.4
2.1	P01‐F02–2.1	18.9	10	90	25	25	1:1 from 2
2.2	P01‐G02–2.2	9.0	10	90	25	25	1:1 from 2.1
2.3	P01‐H02–2.3	5.0	10	90	25	25	1:1 from 2.2
2.4	P01‐A03–2.4	2.5	10	90	25	25	1:1 from 2.3
2.5	P01‐B03–2.5	1.3	10	90	25	25	1:1 from 2.4
3.1	P01‐C03–3.1	20.3	50	50	25	25	1:1 from 3
3.2	P01‐D03–3.2	9.4	50	50	25	25	1:1 from 3.1
3.3	P01‐E03–3.3	5.2	50	50	25	25	1:1 from 3.2
3.4	P01‐F03–3.4	2.6	50	50	25	25	1:1 from 3.3
3.5	P01‐G03–3.5	1.3	50	50	25	25	1:1 from 3.4
4.1	P01‐H03–4.1	20.0	99	1	25	25	1:1 from 4
4.2	P01‐A04–4.2	10.0	99	1	25	25	1:1 from 4.1
4.3	P01‐B04–4.3	5.0	99	1	25	25	1:1 from 4.2
4.4	P01‐C04–4.4	2.4	99	1	25	25	1:1 from 4.3
4.5	P01‐D04–4.5	1.2	99	1	25	25	1:1 from 4.4
5.1	P01‐E04–5.1	19.7	90	10	25	25	1:1 from 5
5.2	P01‐F04–5.2	9.5	90	10	25	25	1:1 from 5.1
5.3	P01‐G04–5.3	5.5	90	10	25	25	1:1 from 5.2
5.4	P01‐H04–5.4	2.6	90	10	25	25	1:1 from 5.3
5.5	P01‐A05–5.5	1.2	90	10	25	25	1:1 from 5.4
6.1	P01‐B05–6.1	20.4	75	25	25	25	1:1 from 6
6.2	P01‐C05–6.2	10.5	75	25	25	25	1:1 from 6.1
6.3	P01‐D05–6.3	5.2	75	25	25	25	1:1 from 6.2
6.4	P01‐E05–6.4	2.6	75	25	25	25	1:1 from 6.3
6.5	P01‐F05–6.5	1.3	75	25	25	25	1:1 from 6.4
7.1	P01‐G05–7.1	19.8	25	75	25	25	1:1 from 7
7.2	P01‐H05–7.2	9.9	25	75	25	25	1:1 from 7.1
7.3	P01‐A06–7.3	5.3	25	75	25	25	1:1 from 7.2
7.4	P01‐B06–7.4	2.8	25	75	25	25	1:1 from 7.3
7.5	P01‐C06–7.5	1.4	25	75	25	25	1:1 from 7.4
8.1	P01‐D06–8.1	40.0	100	0	NA	NA	NA
8.2	P01‐E06‐8.2‐empty	0.0	NA	NA	50	NA	NA

*Note*: Samples were prepared by initially mixing microbial and human DNA in specific ratios (sample names 1–7), followed by a series of dilutions (sample names 1.1–7.5).

Abbreviations: Bacterial, percentage of bacterial DNA in the sample; Comment, comments providing additional context or specific remarks about the sample preparation, such as the source sample for each dilution; Concentration, DNA concentration in ng/µL; H_2_O (µL), volume of DNase/RNase‐free water added for dilution, where applicable; Human, percentage of human DNA in the sample; NA, not applicable; Sample identifier, unique identifier code for each sample; Sample names, descriptive name associated with the sample, including initial samples (1–8) and subsequent dilutions (e.g., 1.1, 1.2); Standard (µL), volume of standard solution added, if any.

#### 5.2.4 Control samples

To ensure robust comparisons and validation, three control samples were prepared:

- Pure microbial DNA (sample name 8): Served as the microbial control.
- Pure human DNA (sample name 8.1): Served as the human control.
- Pure DNase‐ and RNase‐free water (sample name 8.2): Served as the negative control.

All samples were stored at −20 °C until they were shipped to LGC Genomics GmbH (Berlin, Germany) for further analysis.

### 5.3 Digital droplet qPCR quantification

To determine the absolute numbers of 18S and 16S rRNA gene copies in the DNA samples, we used a ddPCR TaqMan assay (Table 4).

**TABLE 4:** Primer and probe sets used in TaqMan digital droplet PCR (ddPCR) assays for 18S and 16S rRNA gene quantification.

Primer set	Forward	Reversed	Probe
16S	281 ACTGAGACACGGCCCA	515 TTACCGCGGCMGCTGGCAC	FAM‐ACTCCTACGGGAGGCAGC‐MGB
18S	387 ACATCCAAGGAAGGCAGCAG	388 TTTTCGTCACTACCTCCCCG	VIC‐CGCGCAAATTACCCACTCCCGAC

Abbreviations: FAM, 6‐carboxyfluorescein (also written as 6‐FAM); MGB, minor groove binder; VIC, VIC® dye (Thermo Fisher Scientific).

#### 5.3.1 Primer and master mix preparation

A 10× concentrated primer‐probe mix was prepared, with final concentrations of 0.8 μmol/L for the primers and 0.4 μmol/L for the probes. The master mix included the QiAcuity demo assay IC probe assay kit yellow (200/50) from QIAGEN (GmbH, Hilden, Germany), the primer‐probe mix, a restriction enzyme, and RNase‐free water. The mixture was vortexed to ensure homogeneity, and 11.8 μL of the master mix was combined with 1 μL of DNA in a 96‐well plate. For each sample, 12 μL of this preparation was transferred to a Nanoplate 8.5 K 96‐well plate (QIAGEN GmbH, Hilden, Germany), with each sample run in duplicate.

#### 5.3.2 PCR cycling conditions

PCR amplification was conducted with the following cycling parameters:

- Initial denaturation: 95 °C for 2 min.
- Amplification: 40 cycles of 95 °C for 15‐s and 60 °C for 30‐s.

Fluorescence detection channels were set to green (FAM) for the 16S rRNA gene and yellow (VIC) for the 18S rRNA gene.

### 5.4 Sample dilution and normalization

To determine the appropriate sample concentrations, various dilutions were tested and evaluated. Final dilutions were as follows:

- 1:100 for samples 4, 5, and 6.
- 1:10,000 for samples 1, 2, 3, 6, 7, and pure bacterial DNA samples.

The final copy numbers were calculated by multiplying the ddPCR output concentration (copies/µL) by the corresponding dilution factor for each sample.

Given the differences in ribosomal gene copy numbers per unit of DNA, reflecting variations in genome size, the values were normalized to a 50:50 ratio, setting both 16 and 18S rRNA genes to a relative value of 1.

### 5.5 DNA sequencing

DNA samples were processed and sequenced by LGC Genomics GmbH (Berlin, Germany). A total of 45 samples were sequenced, yielding approximately 2.5 million reads each. Sequencing was performed using 300‐bp paired‐end reads on the Illumina MiSeq platform (Illumina, Inc., San Diego, CA, USA), targeting the bacterial 16S rRNA gene V3‐V4 region (primers 341F and 785R). Each PCR reaction included 1–10 ng of DNA extract (1 μL), 15 pmol of forward and reverse primers, 1× MyTaq buffer containing 1.5 units of MyTaq DNA polymerase (Bioline GmbH, Luckenwalde, Germany), and 2 μL of BioStabII PCR enhancer (Sigma‐Aldrich Co., St. Louis, MO, USA), in a total volume of 20 μL. The forward and reverse primers included identical 10‐nt barcode sequences for each sample. Amplification was performed over 30 cycles with the following conditions:

- Pre‐denaturation: 96 °C for 1 min.
- Denaturation: 96 °C for 15‐s.
- Annealing: 55 °C for 30‐s.
- Extension: 70 °C for 90‐s.
- Final hold: 8 °C.

The DNA concentration of amplicons was assessed via gel electrophoresis. Approximately 20 ng of amplicon DNA from each sample was pooled for up to 48 samples, each with a unique barcode. Amplicon pools were purified with Agencourt AMPure XP beads (Beckman Coulter, Inc., IN, USA) to remove primer dimers and small mispriming products, followed by additional purification using MiniElute columns (QIAGEN GmbH, Hilden, Germany).

For library construction, 100 ng of each purified amplicon pool was used with the Ovation Rapid DR Multiplex System 1–96 (NuGEN Technologies, Inc., CA, USA). The resulting Illumina libraries were pooled, size‐selected via preparative gel electrophoresis, and sequenced on an Illumina MiSeq platform using V3 chemistry.

All libraries were demultiplexed for each sequencing lane using the Illumina bcl2fastq v2.20 software [37]. The demultiplexing protocol allowed for up to two mismatches or Ns (undetermined bases) in the barcode read, provided that the barcode distances between all libraries on the lane permitted it. After sorting, the barcode sequence was clipped, and reads with missing or conflicting barcodes were removed.

### 5.6 Sequence data processing

Amplicon sequencing data were processed and taxonomically annotated using the LotuS2 pipeline (v2.32) [31, 37, 38]. The SILVA database (version 138) was used as the reference database for taxonomic alignment [24, 39], which was performed using the Lambda aligner [24, 39].

The reference database selected for this process was the SILVA database, specifically version 138 [24, 39], and the taxonomic alignment was conducted using the lambda aligner [40].

To address human contamination in the sequencing data, the LotuS2 (v2.32) pipeline was used with the “offtargetDB” parameter [13, 41, 42] and the human genome version 38 patch 14 (GRCh38.p14), masked using the Progenomes3 database [43]. This approach enabled the removal of OTUs that aligned with the off‐target database after clustering.

#### 5.6.1 Human contamination removal pre‐clustering

Human contamination was removed before clustering using the BBMap tool from BBTools (v38.98) [30]. GRCh38.p14, masked with the Progenomes3 database, was used as the reference. The following parameters were applied for the analysis:

- Minid = 0.95: Minimum identity of 95% between reads and the reference genome.
- Maxindel = 3: Allowed up to three indels (insertions or deletions) in alignments.
- Bwr = 0.16 and bw = 12: Controlled dynamic range and bandwidth for alignment.
- Quickmatch: Enabled rapid identification of matching regions.
- Fast: Optimized the alignment process for efficiency.
- Minhits = 2: Retained only reads with at least two valid hits to the reference genome, further ensuring the removal of human contamination.

#### 5.6.2 Masking and filtering

The GRCh38.p14 human genome was masked using the Progenomes3 database to account for repetitive and low‐complexity regions, thereby improving the accuracy of contamination removal. Reads were classified into “human” and “filtered” categories and stored as separate FASTA files.

#### 5.6.3 Validation of human reads

Human reads were further validated by aligning them to the nt database using BLAST 2.9.0+ [44]. Alignment parameters included:

- Minimum alignment length: 280 bp.
- Percentage identity: ≥95%.
- *E*‐value: <0.01.

### 5.7 Statistical analyses

All statistical analyses were conducted in R (version 4.3.0) using RStudio (version 2023.12.1) [45]. Data included raw, pre‐clustering, and post‐clustering decontaminated datasets, as well as data processed with the Decontam R package (version 1.20.0) [32]. Genus‐level abundance tables were standardized to relative abundances using total sum scaling. For visualization and analysis, only the ten most abundant genera were displayed, with less abundant genera aggregated into an “other” category.

#### 5.7.1 Beta diversity and dimensionality reduction

Beta diversity was assessed using the Bray–Curtis dissimilarity index calculated with the vegdist function (vegan package, version 2.6–4) [46]. PCoA was performed with the cmdscale function (stats package, version 4.3.0) to reduce dimensionality and facilitate interpretation.

#### 5.7.2 PERMANOVA and variance analysis

The influence of DNA ratios and concentrations on beta diversity was evaluated using PERMANOVA with the adonis2 function (vegan package). Variance explained by these factors was quantified and reported.

#### 5.7.3 Visualization and univariate analysis

Figures were generated using the ggplot2 package (version 3.4.2) [47]. Univariate analyses were performed to compare the raw and filtered datasets using the metadeconfound R package (version 0.3.1) [48] to detect potential confounders that may affect microbial abundance estimates.

## AUTHOR CONTRIBUTIONS

**Till Birkner:** Formal analysis; visualization; writing—original draft; writing—review and editing. **Theda Ulrike Patricia Bartolomaeus:** Conceptualization; study design; investigation; visualization; writing—original draft; writing—review and editing. **Victoria McParland:** Investigation; methodology; writing—review and editing. **Sofia Kirke Forslund‐Startceva:** Funding acquisition; writing—review and editing. **Ulrike Löber:** Conceptualization; study design; supervision; project administration; writing—original draft; writing—review and editing. All authors read and approved the final manuscript.

## CONFLICT OF INTEREST STATEMENT

The authors declare no conflicts of interest.

## ETHICS STATEMENT

This study did not involve human participants, human samples requiring individual consent, or animal subjects. The human genomic DNA used in this study was obtained as a commercially available reference material (Promega Corporation, Madison, WI, USA) and was not derived from identifiable individuals or linked to any personal or clinical data. As such, this study did not require additional ethical approval. All procedures performed in studies involving animals were in accordance with the ethical standards of the institution or practice at which the studies were conducted, and with the 1964 Helsinki Declaration and its later amendments, or with comparable ethical standards.

## Supporting information

Supporting Information S1

## Data Availability

The sequencing data supporting the conclusions of this article are publicly available via the sequence read archive (SRA) under accession number PRJNA1268757. All scripts and code used for data processing and analysis are available on the GitHub website (github.com/uloeber/16slowbiomass.git).
