# Supplementary material for: Systematic quantification and removal of host DNA contamination in 16S rRNA gene sequencing
Source: Quant Biol. 2026 Sep 14;14(4):e70055. doi: 10.1002/qub2.70055 (PMC13642109; doi:10.1002/qub2.70055)
Supplement: Supplementary file 1 [file QUB2-14-e70055-s001.docx]

**Supplementary Materials**

**Systematic quantification and removal of host DNA contamination in 16S rRNA gene sequencing**

**Figure S1: Per-sample composition of raw sequencing reads and relative microbial abundance across the bacterial-to-human DNA dilution series.**

(Top) Absolute read counts per individual sample, ordered by bacterial-to-human DNA ratio and dilution step, illustrating raw sequencing depth and variability across replicates. (Bottom) Relative abundance of bacterial genera for each individual sample within the dilution series, including pure bacteria, pure human, and theoretical composition controls. Colors correspond to bacterial genera as indicated in the legend. Unlike Figure 1, which shows composition summarized across replicates, this figure displays each sample individually to illustrate inter-replicate variability underlying the averaged data.

**Figure S2: Absolute abundance of detected taxa across DNA concentration and bacteria-to-human DNA ratio, before and after contamination filtering.**
Absolute abundance (not relative) of bacterial genera detected in naïve (unfiltered), pre-clustering filtered, post-clustering filtered, and Decontam-filtered datasets. (A) Samples ordered by mean absolute DNA concentration (ng/µL). (B) Samples ordered by bacteria-to-human DNA ratio. Colors correspond to bacterial genera as indicated in the legend. This figure complements the relative-abundance analyses presented in the main text (Figures 3–4) by confirming that absolute taxon abundances follow the same overall pattern, supporting the conclusion that relative host DNA content, rather than total DNA concentration, is the primary driver of compositional shifts.

**Figure S3: Relative abundance of genera grouped by bacterial-to-human DNA ratio** for naïve (left), pre-clustering filtered (second), post-clustering filtered (third), and Decontam-filtered (right) datasets. Each column, except "Pure bacteria," depicts the mean relative abundance of the dilution series of the respective ratio. Colors correspond to bacterial genera as indicated in the legend. Unlike the pre-clustering and post-clustering filtering approaches, Decontam filtering did not remove noHit reads (brown), i.e., non-bacterial, host-derived sequences: the proportion of noHit reads in the Decontam-filtered panel remains comparable to the naïve (unfiltered) dataset, particularly in samples with low bacterial content (e.g., 1:99). This illustrates the reduced effectiveness of Decontam at removing host DNA contamination in low-biomass samples relative to the other tested methods.
